# Antiplatelet use in all-cause dementia and dementia subtypes among older adults: a population-based retrospective study

**DOI:** 10.3325/cmj.2026.67.297

**Published:** 2026-08

**Authors:** Zehra Sarikaya Demirbas, Mehmet Ilkin Naharci

**Affiliations:** Department of Geriatrics, Gulhane Faculty of Medicine and Gulhane Training and Research Hospital, University of Health Sciences, Ankara, Türkiye

## Abstract

**Aim:**

To evaluate the appropriateness of antiplatelet use across dementia subtypes in older adults and to identify factors associated with inappropriate antiplatelet use.

**Methods:**

This population-based retrospective study enrolled adults aged ≥65 years with dementia evaluated at a tertiary geriatric outpatient clinic at Gulhane Training and Research Hospital between 2016 and 2025. Antiplatelet (aspirin or clopidogrel) use was categorized as appropriate use, underuse, overuse, or appropriate non-use according to the 2021-2024 European Society of Cardiology guidelines. Multivariable logistic regression analyses were performed to identify the factors associated with inappropriate antiplatelet use.

**Results:**

Among 683 older adults with dementia, 40.7% received antiplatelet therapy. Antiplatelet underuse and overuse were observed in 16.8% and 16.1% of the participants, respectively. Underuse was most prevalent in vascular dementia (38.9%), whereas overuse was most prevalent in Alzheimer disease (20.2%) and frontotemporal dementia (19.0%). In multivariable analyses, antiplatelet overuse was independently associated with diabetes mellitus (odds ratio [OR] 4.31; 95% confidence interval [CI] 1.66-11.16) and inversely associated with current smoking (OR 0.06; 95% CI 0.06-0.67). Antiplatelet underuse was independently associated with female sex (OR 4.44; 95% CI 1.52-12.92).

**Conclusions:**

Our findings highlight the gaps between guideline recommendations and real-world practice and support the need for individualized dementia-sensitive antiplatelet treatment strategies.

Clinical trial registration number: NCT07313137

As the population ages worldwide, dementia is becoming a major clinical and public health problem (1). Dementia affects more than 55 million older adults, with nearly 10 million new cases diagnosed each year, which places it among the leading causes of mortality globally (2). Dementia syndromes primarily arise from neurodegenerative conditions: Alzheimer disease (AD), dementia with Lewy bodies (DLB), Parkinson disease dementia (PD), and vascular dementia (VaD) due to cerebrovascular diseases (3,4). Increasing evidence demonstrates that vascular pathology exerts an independent additive effect on the progressive loss of neurons in neurodegenerative conditions (5).

The coexistence of neurodegenerative and vascular dementia syndromes with substantial cerebrovascular pathology highlights the intertwined relationship between vascular injury and cognitive impairment (6). Although AD is primarily characterized by amyloid-β and tau deposition, microvascular abnormalities (including cerebral amyloid angiopathy, neurovascular unit dysfunction, and small-vessel disease) also significantly affect its onset and progression by exacerbating neuronal damage and reducing cerebral perfusion (5,7). Similarly, DLB and PD often occur in the presence of vascular risk factors and white-matter ischemic changes, which modify cognitive trajectories and increase susceptibility to neurodegeneration (8,9). In contrast, VaD is mostly driven by ischemic infarcts, chronic hypoperfusion, and widespread small-vessel pathology; yet its frequent overlap with neurodegenerative proteinopathies supports the concept of mixed dementia as a continuum rather than a distinct diagnostic entity (6). This shared vascular vulnerability suggests that approaches targeting vascular pathology, including the judicious use of antiplatelet agents, may play a meaningful role in improving clinical outcomes in different dementia subtypes.

Considering the role of vascular pathology in cognitive decline, antiplatelet therapy may mitigate the risk of dementia in individuals with cerebral small-vessel disease by slowing the underlying vascular pathophysiological processes (10). Although observational studies suggest that antiplatelet drugs can attenuate cognitive deterioration in populations with a high vascular burden, these findings have not been consistently corroborated in randomized clinical trials (11,12). Moreover, substantial gaps persist in the literature regarding antiplatelet prescribing patterns across distinct dementia subtypes and the extent to which such practices align with current clinical guidelines (13,14). Notably, real-world evidence examining how vascular risk profiles influence prescribing behaviors and appropriateness remains limited in the population with dementia.

The aims of this study were to evaluate the frequency of antiplatelet use in older adults with different dementia subtypes and to examine the clinical appropriateness of such use, along with the factors influencing it. By integrating comprehensive clinical, cognitive, and vascular assessments, we sought to determine the extent to which antiplatelet use aligns with the 2021-2024 European Society of Cardiology (ESC) recommendations.

## Participants and methods

### Study design and sample

**Figure 1:**
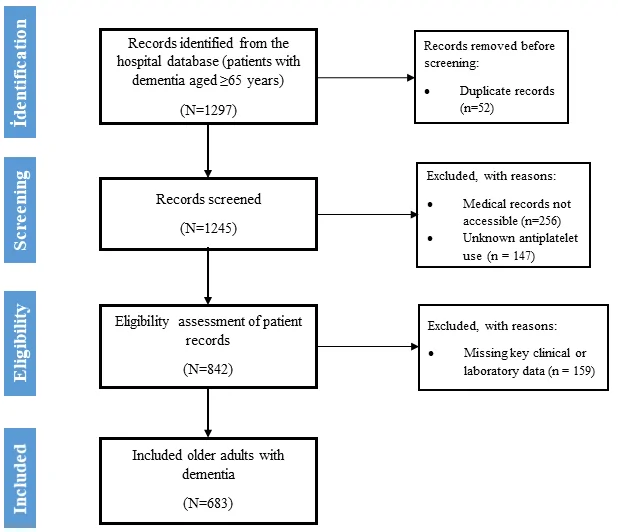
The flowchart of sample selection

We reviewed the medical records of 1297 adults with dementia aged ≥65 years who were evaluated at a tertiary-level geriatric outpatient clinic of Gulhane Training and Research Hospital from 2016 to 2025. Data were gathered from hospital electronic medical records, archived patient files, and standardized comprehensive geriatric assessment (CGA) records. Individuals with inaccessible medical records, unknown antiplatelet use, or missing clinical data were not included in the study (Figure 1). The study was conducted in accordance with the ethical principles of the Declaration of Helsinki. Ethical approval was obtained from the Gulhane Training and Research Hospital Ethics Committee. Written informed consent was not required, as the study was based on a retrospective review of hospital records and all relevant ethical standards were adhered to. The reporting of this study followed the PROBE checklist where applicable. The index date for each participant was defined as the date of their admission to the geriatric outpatient clinic. At the index visit, participants underwent a standardized CGA performed by a geriatrician. The CGA included the assessment of sociodemographic characteristics, clinical findings, blood pressure measurements, dementia severity using the Clinical Dementia Rating (CDR) scale, and laboratory parameters. Information regarding chronic comorbidities, previous cardiovascular and cerebrovascular diseases, medication history, and major bleeding history was extracted from the hospital's electronic medical records and archived patient files and, whenever available, verified using outpatient clinical records, current medication lists, and information obtained from patients or their caregivers.

### Definitions of dementia

At our institution, dementia was diagnosed at the index outpatient visit through a joint clinical evaluation by two senior geriatricians. They used the diagnostic criteria that were in effect at the time (DSM-IV or DSM-5, as applicable) (15,16). Dementia subtypes were classified according to the internationally recognized diagnostic criteria. AD was diagnosed based on the National Institute on Aging-Alzheimer Association 2011 guidelines (17); VaD according to the NINDS-AIREN criteria 1993 (18); DLB according to the 2005 and updated 2017 International DLB Consortium criteria (19,20); and frontotemporal dementia (FTD) according to the consensus clinical diagnostic criteria by Neary et al (21). Mixed dementia was defined as the co-occurrence of at least two different dementia subtypes (22).

### Antiplatelet drug status

Antiplatelet therapy appropriateness was evaluated according to the 2021 and 2024 ESC guidelines available at the time of data analysis. We determined whether antiplatelet therapy at the index outpatient visit was consistent with the most up-to-date evidence-based recommendations. The ESC guidelines were selected as a standardized evidence-based reference to ensure an objective and consistent assessment of treatment appropriateness across the study population (13,14). Aspirin was considered appropriate for secondary prevention in individuals with established atherosclerotic cardiovascular disease (ASCVD), whereas clopidogrel was regarded as an alternative for patients with aspirin intolerance (13). Additionally, the preferential use of clopidogrel over aspirin in certain patients with established ASCVD was also considered acceptable. According to the ESC 2021 guideline, secondary prevention in patients with non-cardioembolic ischemic stroke or transient ischemic attack may be achieved with aspirin alone, aspirin plus dipyridamole, or clopidogrel alone (13). Dipyridamole, ticagrelor, and prasugrel were excluded from the analysis because their use is primarily recommended for specific clinical settings, including acute coronary syndromes, short-term dual antiplatelet therapy, and selected cerebrovascular indications (23-25). Therefore, evaluating the safety and tolerability of different antiplatelet agents was beyond the scope of this study. In addition, the appropriateness of antiplatelet therapy in older adults should be individualized according to the patient's clinical characteristics, bleeding risk, and treatment goals (26). Beyond secondary prevention, considerations for primary prevention were also assessed. With respect to primary prevention, the ESC 2021 guidelines do not recommend antiplatelet therapy in the general population; therefore, antiplatelet use in patients without a clear primary prevention indication was considered inappropriate (13,14).

The present study focused on chronic and stable antiplatelet therapy patterns, specifically examining aspirin (75-100 mg/d) and clopidogrel (75 mg/d). Current antiplatelet drug use at the index outpatient visit was defined as the continuous use of aspirin or clopidogrel for at least 3 months. Accordingly, patients who had completed a short-term course of dual antiplatelet therapy and were receiving long-term aspirin or clopidogrel monotherapy at the index outpatient visit were classified according to their current chronic antiplatelet regimen. Antiplatelet therapy use patterns were classified into four categories according to the ESC 2021-2024 recommendations: “appropriate use,” defined as antiplatelet therapy prescribed for established secondary prevention indications (eg, previous myocardial infarction, ischemic stroke, transient ischemic attack, or other documented ASCVD) in accordance with current ESC guidelines; “underuse,” defined as an absence of antiplatelet therapy in patients for whom the treatment was strongly recommended; “overuse,” defined as the prescription of antiplatelet agents without a guideline-supported indication, thereby exposing patients to unnecessary bleeding risk without expected cardiovascular benefit; and “appropriate non-use,” defined as the absence of antiplatelet therapy in patients without a valid indication (27).

### Variables

Age, sex, and current smoking status were recorded. Arterial blood pressure was measured in the right arm after 5-10 minutes of rest, with patients either in a seated or supine position (28). Global CDR scores, calculated using a standardized algorithm, were used to assess the severity of dementia. Information was obtained through semi-structured interviews with patients and informants. Cognitive-functional status was evaluated across six domains: memory, orientation, judgment and problem-solving, community affairs, home and hobbies, and personal care. Based on these assessments, participants were classified as having mild (CDR = 1), moderate (CDR = 2), or severe (CDR = 3) dementia (29). Major bleeding was defined according to internationally accepted criteria as the presence of any of the following: a reduction in hemoglobin of ≥2 g/dL, the requirement for transfusion of at least two units of packed red blood cells, bleeding occurring in a critical anatomical site (eg, intracranial, intraspinal, intraocular, retroperitoneal, intra-articular, pericardial, or intramuscular with compartment syndrome), or any hemorrhagic event leading to hemodynamic instability requiring medical intervention (30).

The chronic comorbidities evaluated in this study were hypertension, cardiovascular disease, diabetes mellitus, chronic kidney disease (defined as an estimated glomerular filtration rate <60 mL/min/1.73 m^2^), non-cardioembolic ischemic stroke or transient ischemic attack, Parkinson disease, and chronic obstructive pulmonary disease. Data on these conditions were obtained from patients or caregivers and confirmed through current medication lists and detailed examination of available clinical records. Laboratory assessments included serum glucose, total cholesterol, low-density lipoprotein (LDL) cholesterol, and high-density lipoprotein (HDL) cholesterol measurements.

### Statistical analysis

Categorical data are summarized as counts and percentages, while continuous data are summarized as means with standard deviations or medians with ranges. Differences in categorical variables were assessed with a χ^2^ test. The distribution of continuous variables was assessed with a Kolmogorov-Smirnov test, and differences in continuous variables were assessed with one-way ANOVA. Logistic regression analysis was performed to examine the association between appropriate antiplatelet use (dependent variable) and age, sex, dementia severity, history of major bleeding, hypertension, diabetes mellitus, chronic kidney disease, Parkinson disease, HDL cholesterol, and LDL cholesterol (independent variables). The multivariable model included variables that were statistically significant in univariate analyses and those considered clinically relevant. In addition, binary logistic regression models were constructed to identify the predictors of underuse and overuse, employing appropriate use as the reference category. Results were expressed as odds ratios (ORs) with corresponding 95% confidence intervals (CIs). The level of statistical significance was defined as a two-tailed *P* value <0.05. Model calibration was evaluated using the Hosmer-Lemeshow goodness-of-fit test, and the overall model adequacy was assessed with the omnibus test. All findings were interpreted within the framework of a 95% CI. Statistical analysis was performed with SPSS, version 29.0 (IBM Corp, Armonk, NY, USA).

## RESULTS

The study enrolled 683 older adults (61.2% women), with a mean age of 79.1 ± 6.5 years. Overall, 278 individuals (40.7%) used antiplatelet therapy. The proportion of women was higher among antiplatelet non-users than among users (64.7% vs 56.1%; *P* = 0.015), and current smoking was more common among antiplatelet users (11.8% vs 5.4%, *P* = 0.024). Most participants had moderate (43.5%) or mild (41.1%) dementia, followed by severe dementia (15.4%). Mild dementia was more frequent among antiplatelet users, whereas moderate and severe dementia were more prevalent among non-users; however, the dementia stage did not differ significantly between the groups (*P* = 0.586). A total of 13.7% of participants had a history of major bleeding, with this condition being significantly more prevalent among non-users (18.8% vs 6.0%, *P* < 0.001).

The most prevalent comorbidity was hypertension (59.3%), followed by cardiovascular disease (33.2%), diabetes mellitus (31.8%), and chronic kidney disease (22.4%). Participants with hypertension, cardiovascular disease, diabetes mellitus, and non-cardioembolic ischemic stroke or transient ischemic attack were more likely to use antiplatelet therapy (all *P* ≤ 0.001). The groups did not differ significantly in age, systolic blood pressure, chronic kidney disease, Parkinson disease, chronic obstructive pulmonary disease, and laboratory data (all *P* > 0.05) (Table 1).

**Table 1:** Characteristics of the study group*^†^

Variables	Antiplatelet drug use		
	total (N = 683)	yes (n = 278)	no (n = 405)
Age (years), mean ± SD	79.1 ± 6.5	79.5 ± 6.8	79.1 ± 6.3
Sex (female), n (%)	418 (61.2)	156 (56.1)	262 (64.7)
Smoking (current), n (%)	32 (8.0)	19 (11.8)	13 (5.4)
Systolic blood pressure (mmHg), mean ± SD	131.6 ± 17.9	132.1 ± 18.1	131.6 ± 17.0
Dementia severity, n (%)			
mild	281 (41.1)	120 (43.2)	161 (39.8)
moderate	297 (43.5)	119 (42.8)	178 (44.0)
severe	105 (15.4)	39 (14.0)	66 (16.2)
Major bleeding history, n (%)	76 (13.7)	13 (6.0)	63 (18.8)
Chronic diseases, n (%)			
hypertension	405 (59.3)	195 (70.1)	210 (51.9)
cardiovascular disease	227 (33.2)	137 (49.3)	90 (22.2)
diabetes mellitus	217 (31.8)	107 (38.5)	110 (27.2)
chronic kidney disease	153 (22.4)	72 (25.9)	81 (20.0)
non-cardioembolic ischemic stroke or	TIA 96 (14.1)	56 (20.1)	40 (9.9)
Parkinson disease	47 (6.9)	22 (7.9)	25 (6.2)
COPD	47 (6.9)	24 (8.6)	23 (5.7)
Laboratory assessments (mg/dL), median (min-max)			
glucose	105 (120-405)	104 (61-405)	105 (71-205)
total cholesterol (mg/dL),	195 (83-370)	197.5 (83-372)	194 (86-370)
LDL cholesterol	118 (34-269)	117 (33-269)	118 (19-246)
HDL cholesterol	48 (19-97)	48 (18-97)	48 (24-91)

*Abbreviations: COPD – chronic obstructive pulmonary disease, LDL – low-density lipoprotein; HDL – high-density lipoprotein; TIA – transitory ischemic attack.

†Missing data: smoking (n = 282), major bleeding history (n = 129), glucose (n = 81), lipid panel (n = 106).

Most of participants (42.5%, n = 290) appropriately did not use antiplatelets, while 24.6% (n = 168) used them appropriately. Inappropriate antiplatelet use was also common (32.9%), with underuse observed in 16.8% (n = 115) and overuse in 16.1% (n = 110) of participants.

Appropriate antiplatelet use was most frequent among patients with DLB (44.4%) and mixed dementia (44.4%), followed by those with VaD (40.3%). Appropriate non-use predominated in AD (55.8%) and DLB (52.4%). Regarding inappropriate use, underuse was particularly prevalent in VaD (38.9%), whereas overuse was most commonly observed in AD (20.2%) and FTD (19.0%) (Table 2).

**Table 2:** Appropriateness of antiplatelet drug use across dementia subtypes

Appropriateness of antiplatelet drug use	Total (N = 683)	Dementia subtypes; No. (%)					
		Alzheimer disease (n = 380)	vascular dementia (n = 144)	dementia with Lewy bodies (n = 63)	frontotemporal dementia (n = 21)	Parkinson disease dementia (n = 12)	mixed dementia (n = 63)
Appropriate use	168 (24.6)	47 (12.4)	58 (40.3)	28 (44.4)	4 (19.0)	3 (25.0)	28 (44.4)
Underuse	115 (16.8)	44 (11.6)	56 (38.9)	1 (1.6)	3 (14.3)	3 (25.0)	8 (12.7)
Overuse	110 (16.1)	77 (20.2)	15 (10.4)	1 (1.6)	4 (19.0)	2 (16.7)	11 (17.5)
Appropriate non-use	290 (42.5)	212 (55.8)	15 (10.4)	33 (52.4)	10 (47.7)	4 (33.3)	16 (25.4)

**Figure 2:**
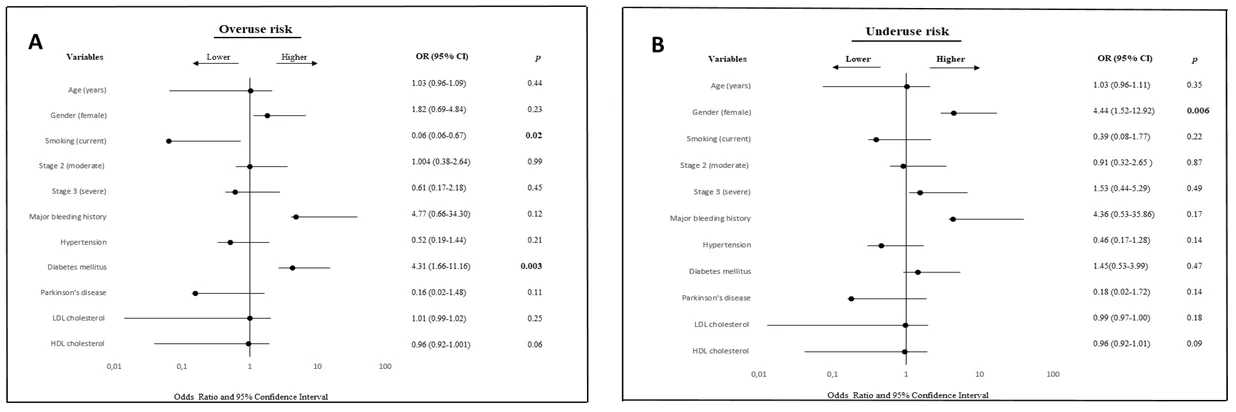
Factors associated with antiplatelet overuse (**A**) and underuse (**B**). Odds ratios are presented with 95% confidence intervals derived from binary logistic regression models. Statistically significant predictors are shown in bold.

In the multivariable binary logistic regression analysis, antiplatelet overuse was independently significantly associated with diabetes mellitus (OR 4.31; 95% CI 1.66-11.16; *P* = 0.003) and inversely associated with current smoking (OR 0.06; 95% CI 0.06-0.67; *P* = 0.02) (Figure 2A). In addition, antiplatelet underuse was independently significantly associated with female sex (OR 4.44; 95% CI 1.52-12.92; *P* = 0.006) (Figure 2B).

For the antiplatelet overuse model, the Hosmer-Lemeshow goodness-of-fit test was non-significant (χ^2^ = 8.001; *P* = 0.433), indicating an adequate model fit, while the omnibus test indicated the significance of the model (−2LL = 123.903; χ^2^ (2) = 25.031; *P* = 0.009). For the antiplatelet underuse model, the Hosmer-Lemeshow test was non-significant (χ^2^ = 2.983; *P* = 0.935), and the omnibus test demonstrated overall model significance (−2LL = 121.887; χ^2^ (2) = 20.765; *P* = 0.036).

The sensitivity analyses yielded results that were largely consistent with the primary analyses. After excluding participants with a history of major bleeding, antiplatelet overuse remained independently associated with diabetes mellitus (OR 3.01; 95% CI 1.32-6.81; *P* = 0.008) and inversely associated with current smoking (OR 0.19; 95% CI 0.04-0.94; *P* = 0.042). In contrast, no independent variables were significantly associated with antiplatelet underuse. The absence of unnecessary restrictions on antiplatelet therapy in patients without a history of major bleeding suggests that this finding is clinically expected and consistent.

## Discussion

In our study, approximately half of the participants (41%) used antiplatelet therapy. One-third used antiplatelets inappropriately: half of these participants were underusers and half were overusers. Notably, antiplatelet underuse was most prevalent among individuals with VaD, whereas overuse was most common in neurodegenerative dementia subtypes. Also, diabetes mellitus was an independent predictor of antiplatelet overuse, while female sex was independently linked to a higher likelihood of underuse. Our results provide additional findings on antiplatelet use in older adults with dementia, having a potential to guide appropriate prescribing processes in this vulnerable population and reduce the risk of bleeding side effects due to overuse.

To our knowledge, no population-based study has quantitatively evaluated the prevalence of antiplatelet use in this population, which limits the direct comparability of our findings. Studies conducted in inpatient and outpatient care settings have reported mixed results. Resnick et al (26) reported that approximately 60% of hospital-based patients were using aspirin and/or clopidogrel. However, this finding reflects the high cardiovascular burden and acute clinical indications specific to inpatient populations rather than deliberate prescribing decisions in stable outpatient settings (26). In nursing home residents with dementia, Tjia et al (31) reported antiplatelet use below 10%. In the context of this study, aspirin was deliberately excluded, and the care approach was comfort-oriented and based on deprescribing principles, which limited the study’s generalizability to non-palliative populations (31). In a substudy of the IDEAL study, antiplatelet agents were among the most frequently identified potentially inappropriate drugs in individuals with advanced dementia; yet, this analysis focused on medication burden and end-of-life prescribing practices rather than on guideline adherence or clinical indications (32). Notably, Michalowsky et al (33) reported antiplatelet use by 20.1% of dementia patients in primary care. However, the study population was heterogeneous in terms of recruitment settings and referral pathway, encompassing various disease stages and clinical profiles, which complicates direct inferences regarding prescribing appropriateness (33). Owing to the inclusion of a homogeneous study population, our research may serve as a reference for future research conducted in different cohorts of community-dwelling older adults with dementia.

Our study revealed considerable inappropriate antiplatelet use in our study population and a distinct variation of the prescribing pattern according to the dementia subtype. The overuse pattern identified in neurodegenerative dementias is consistent with the findings of a Brazilian cross-sectional study among older adults with AD, in which cardiovascular medications, especially acetylsalicylic acid, were among the most commonly identified inappropriate drugs (34). In contrast, the underuse of antiplatelet therapy in VaD may reflect clinical hesitancy driven by concerns regarding bleeding risk, frailty, and prognostic uncertainty. This interpretation is further supported by the findings of Viticchi et al (35), who argued that frequent suboptimal management of atrial fibrillation in patients with VaD potentially contributed to the insufficient implementation of effective cardiovascular treatment strategies in this population (35). Taken together, the variation in prescribing pattern underscores that, beyond the general challenges of translating guideline recommendations into clinical practice, dementia subtype and cognitive status play critical roles in shaping antiplatelet prescribing decisions among individuals with dementia.

The association between female sex and antiplatelet underuse observed in our analysis is consistent with previous studies reporting sex-related disparities in cardiovascular preventive care (36). These findings suggest that sex-based differences in antiplatelet therapy prescription vary across health care systems and clinical practice settings. Moreover, the association between diabetes mellitus and antiplatelet overuse is expected and clinically plausible. The perception of diabetes mellitus as a surrogate for “high vascular risk” may contribute to the continuation of aspirin or other antiplatelet therapy without reassessment of the indication, particularly in the context of primary prevention. Supporting this interpretation, observational data from older populations in the United States have shown that diabetes mellitus is associated with increased use of aspirin for primary prevention (37). In addition, the literature findings on the association between current smoking and antiplatelet overuse are inconsistent. While some real-world cardiology cohorts have reported smoking to be associated with a higher likelihood of aspirin use, other studies have failed to demonstrate a consistent relationship (38). This heterogeneity may reflect differences in cardiovascular risk stratification, comorbidity burden, and individualized clinical decision-making among the study populations.

This study has some limitations. The retrospective, single-center design necessitates that the findings be interpreted as reflecting real-world observational associations rather than causal relationships; however, the detailed data derived from the comprehensive geriatric assessment and the systematic classification based on contemporary ESC guideline recommendations support the clinical relevance and applicability of the results. The appropriateness of antiplatelet therapy was assessed only at the index outpatient visit, and no longitudinal changes in prescribing and deprescribing decisions were evaluated. Although antiplatelet use was determined through detailed review of medical records, some members of the overuse group might have been taking over-the-counter antiplatelets. Nevertheless, the consistency of the findings in sensitivity analyses excluding individuals with a history of major bleeding strengthens the robustness of the results. Moreover, to enhance data reliability, all cases were evaluated through a structured and standardized assessment process conducted by an experienced multidisciplinary geriatric team. Finally, to minimize selection bias, participants were enrolled consecutively and chronologically according to their first admission to the geriatric outpatient clinic, thereby ensuring a representative real-world cohort.

In conclusion, our findings highlight the importance of routinely reassessing the indications for antiplatelet therapy in older adults with dementia, rather than continuing treatment solely based on historical prescribing decisions. Particular attention should be paid to patients with VaD, in whom underuse may compromise secondary cardiovascular prevention, and to those with neurodegenerative dementias, in whom unnecessary antiplatelet therapy may increase the bleeding risk without providing a clear clinical benefit. Therefore, incorporating regular medication reviews into comprehensive geriatric assessments may therefore facilitate more individualized, guideline-concordant prescribing in this vulnerable population. These findings underscore the need for more individualized, dementia-sensitive approaches to antiplatelet drug use.
